# Calibrated Emotional Engagement in Perioperative Care: A Middle‐Range Theory for Sustainable Clinical Practice

**DOI:** 10.1111/nin.70122

**Published:** 2026-06-09

**Authors:** Johan Eriksson

**Affiliations:** ^1^ Department of Diagnostics and Intervention Umeå University Umeå Sweden

**Keywords:** calibrated emotional engagement, emotion regulation, emotional labour, empathy, middle‐range theory, operating room, perioperative nursing, workforce sustainability

## Abstract

Emotional engagement in clinical practice has been theorised as a professional virtue, a form of emotional labour and a potential pathway to compassion fatigue and burnout. Although these traditions have shown that emotional practice is shaped by relational and organisational conditions, they have less clearly specified how clinicians regulate engagement in high‐demand perioperative settings, or what organisational conditions make such regulation achievable. Drawing on emotion regulation theory, nursing scholarship on caring and emotional labour and Donabedian's structure–process–outcome framework, we introduce calibrated emotional engagement as a middle‐range theoretical concept describing the dynamic, professionally regulated adjustment of emotional, attentional and relational involvement required to remain clinically responsive without becoming detached or overwhelmed. Calibrated emotional engagement is understood as a multilevel process: individually enacted, team‐mediated and organisationally conditioned. A three‐zone model, comprising under‐engagement, over‐engagement and a calibrated zone of functionally sustainable engagement, offers a way of conceptualising the range of emotional states in high‐demand perioperative settings, including the cue‐deprived character of the operating room where anaesthetised patients and sterile draping remove the relational feedback that ordinarily supports empathic practice. We further propose that organisational conditions, including staffing, team stability, autonomy and recovery structure, constitute an empathic infrastructure that shapes whether calibrated engagement is achievable in practice. The concept opens new directions for perioperative education, workforce policy and research that move beyond individual resilience towards systemic accountability for the emotional conditions of care.

## 1 Introduction: The Invisible Labour of the Operating Room

Something is consistently absent from accounts of perioperative practice. The safety literature attends to checklists, handovers and communication failures. The workforce literature counts hours, vacancies and attrition rates. The quality literature tracks infection rates, surgical outcomes and patient satisfaction scores. What these frameworks share is a studied inattention to the emotional dimension of perioperative work, to the labour of managing one's own feeling states in an environment that generates them continuously and intensely.

This inattention is not accidental. Emotional labour in clinical practice occupies an ambiguous status: valorised in nursing theory as empathy, compassion and presence (Watson 1988; Benner and Wrubel 1989), yet persistently marginalised in the systems‐level frameworks that determine how care is organised, staffed and evaluated. Huynh et al. (2008) describe emotional labour as a central but chronically underrecognised component of nursing practice, and Delgado et al. (2017) demonstrate that its costs, when unaddressed, accumulate directly into emotional exhaustion and attrition. When emotional engagement appears in the perioperative literature at all, it clusters around two poles. The first is normative: empathy is a professional virtue, a core component of patient‐centred care, something clinicians should cultivate and authentically express. The second is risk‐oriented: sustained emotional engagement is a source of occupational burden, a pathway to compassion fatigue and burnout (Figley 1995; Maslach 2001). Both framings share a consequential flaw: they treat emotional engagement as primarily an individual matter rather than as a practice shaped by the conditions of work.

This characterisation does not ignore scholarship that has already moved in a structural direction. A substantial body of work has shown that emotional labour is shaped by relational, organisational and institutional conditions (Doane and Varcoe 2015; Riley and Weiss 2016), and that work environment factors are linked to both clinician experience and patient outcomes (Aiken et al. 2012; Gunnarsdóttir et al. 2009). What has remained less clearly specified, however, is the regulatory process through which clinicians maintain functional emotional engagement under the particular demands of perioperative practice, and how that process is enabled or constrained by specific organisational conditions. It is this process‐level gap, not the observation that structure matters, which is already well‐established, that the present model attempts to address.

This paper proposes a different analytical frame. We introduce calibrated emotional engagement as a middle‐range theoretical concept that reconceptualises emotional regulation in perioperative care, not as a personal trait or a moral achievement, but as a professionally cultivable and organisationally conditioned practice. The concept enables a shift from asking ‘how empathic is this nurse?’ to asking ‘what conditions make calibrated engagement achievable, and which foreclose it?’

The operating room is not simply an intensified version of other clinical environments; it is structurally different in ways that reshape the clinician‐patient relationship at its foundation. Most notably, it may be characterised as a cue‐deprived empathy context: the patient is typically anaesthetised and visually bounded by sterile draping, removing the real‐time relational feedback, including facial expression, verbal communication and bodily presence, that ordinarily scaffolds empathic engagement (Nortvedt 2001). The OR may thus require clinicians to sustain a cognitive representation of the person beneath the drapes through professional attention alone, in the absence of the intersubjective cues that make this effortless elsewhere. This distinguishes it from other settings involving unconscious patients, such as intensive care, where extended contact, family presence and narrative continuity provide alternative relational anchors. Team familiarity and communicative continuity, both of which are often disrupted in contemporary perioperative staffing models, may further compound this challenge (Frasier et al. 2019).

The argument proceeds by first positioning the concept in relation to adjacent frameworks, then specifying the process of calibration, developing the three‐zone model, and identifying the organisational conditions that shape whether calibrated engagement is achievable in practice. We conclude with implications for education, policy and research, and with a restatement of the paper's central claim: that sustainable emotional regulation in the operating room is substantially a structural problem, and warrants structural attention.

## 2 The Limits of Existing Frameworks

The concept of empathy has a long and contested history in nursing theory. From Watson (1988) caring science to Benner and Wrubel's (1989) account of skilled caring practice, the dominant emphasis has been on cultivating emotional responsiveness as a moral disposition, a character orientation to be developed through education and reflection. This tradition has generated important nursing scholarship, and its insistence that caring is substantive professional work, not incidental sentiment, remains valuable. What it has not provided is an account of how emotional engagement should be regulated under conditions of high demand, compressed time and procedural intensity. It offers a vision of ideal practice without a theory of how that practice is sustained.

The emotional labour tradition, originating with Hochschild (1983) account of the commercial management of feeling and adapted for nursing by James (1992) and Smith (1992), provides a more critical analytical purchase. By foregrounding the work involved in managing feeling states, and situating this work within the labour relations of care, this tradition exposes what normative accounts conceal: that emotional engagement is not freely given but extracted, often under conditions of insufficient recognition. The emotional labour framework makes visible the systemic production of emotional cost in caring work.

Importantly, more recent scholarship within this tradition has explicitly situated emotional labour within relational, organisational and sociomaterial contexts (Doane and Varcoe 2015; Riley and Weiss 2016), and sociological accounts of healthcare work have foregrounded institutional norms, power relations and professional hierarchies as constitutive of emotional practice (Allen 2014). What this tradition has been less consistently developed to specify is what functional, sustainable emotional regulation looks like within those systemic conditions, and how the regulatory process itself might be theorised and supported.

The contemporary distinction between affective empathy, understood as sharing another's emotional state, and cognitive empathy, understood as representing another's perspective without necessarily sharing their affect, adds a further dimension (Decety and Jackson 2004). In high‐demand, procedurally intensive environments, full affective resonance with each patient's fear, pain, or vulnerability is neither sustainable nor, in many circumstances, functionally adaptive. The surgeon absorbed in her patient's anxiety will not perform better. The nurse who carries the emotional weight of every induction across a full operating list will not last a career. Yet the normative tradition has been slow to integrate this distinction, and educational frameworks continue to treat empathy as a global disposition to cultivate rather than a multidimensional capacity to regulate.

The burnout literature, despite its empirical richness, similarly tends towards an individual‐centred intervention logic. Maslach (2001) model identifies emotional exhaustion, depersonalisation and reduced personal accomplishment as the dimensions of burnout in helping professions. Figley (1995) compassion fatigue framework focuses on cumulative exposure and individual coping capacity. These frameworks are not wrong; they describe real phenomena with real consequences. But they are reactive: they trace what happens when emotional regulation fails, not what functional regulation looks like or how its structural preconditions might be designed. The implicit resolution is typically individual‐level, centred on building resilience, developing coping skills and accessing support services. What is missing is a positive, mechanistic account of regulated emotional engagement that is also structurally grounded.

Recent nursing scholarship has begun to conceptualise and empirically examine emotional regulation as a significant dimension of nursing practice. Fasbinder et al. (2020) analyse emotional regulation as a nursing concept, mapping its attributes and demonstrating its relevance to professional functioning. Feng et al. (2024) conduct a scoping review of emotional labour levels and outcomes in nurses, identifying links between emotional labour, organisational conditions and workforce‐related outcomes. Erkayiran and Demirkiran (2024) demonstrate in a quasi‐experimental study that emotion regulation training may positively influence nurses' emotional wellbeing and professional functioning. The present paper does not claim that emotional regulation in nursing is an unexplored phenomenon. Its contribution is more specific: to theorise how emotional, attentional and relational regulation is enacted under the distinctive conditions of perioperative practice, and how this process is shaped by staffing, team stability, autonomy and recovery structure. Calibrated emotional engagement is therefore proposed not as a replacement for existing emotional regulation or emotional labour frameworks, but as a perioperative middle‐range specification of how regulated engagement may be sustained, disrupted and organisationally supported.

## 3 Introducing Calibrated Emotional Engagement

We propose the following definition

Calibrated emotional engagement refers to the dynamic, professionally regulated adjustment of emotional, attentional, and relational involvement required to remain clinically responsive without becoming either detached or overwhelmed in high‐demand care environments.

The term calibrated is chosen deliberately. Calibration implies precision, continuous adjustment to changing conditions, and a process that must be actively performed rather than a state that is possessed. Crucially, it implies that the instrument of calibration can be trained and maintained and can be rendered more or less reliable by the conditions in which it operates. This last implication is the conceptual core of the model: calibration may be understood not only as an individual skill but as an organisationally conditioned capacity.

Calibrated emotional engagement is therefore best understood as a multilevel process: individually enacted, team‐mediated and organisationally conditioned. At the individual level, it draws on regulatory capacity, professional identity and accumulated clinical experience. At the team level, it is shaped by psychological safety, communicative continuity and collective norms around emotional expression and support. At the organisational level, it is enabled or constrained by staffing, structure and the design of recovery opportunities. These levels are not independent; organisational conditions shape the resources available to teams and individuals, while individual and team practices constitute the micro‐processes through which structural influences are realised or resisted. This multilevel framing is necessary to avoid reproducing the individualisation the model aims to critique while also avoiding a structural determinism that leaves no room for professional agency.

### 3.1 Conceptual Positioning

Table 1 summarises the positioning of calibrated emotional engagement in relation to adjacent constructs. The model does not aim to replace these traditions; it draws on each while directing attention to what each leaves underdeveloped: the specification of the regulatory process through which functional engagement is maintained under perioperative demand, and the organisational conditions that shape whether such regulation is achievable.

**Table 1:** Conceptual positioning of calibrated emotional engagement (CEE) in relation to adjacent constructs.

Construct	What it explains	Primary limitation	What CEE adds
Caring/empathy (Watson; Benner & Wrubel)	Responsiveness to another's experience as a moral disposition	Treats empathy as virtue; no theory of regulation under demand	Specifies functional calibration rather than ideal disposition
Emotional labour (Hochschild; James; Smith)	The work of managing feeling states within labour relations of care	Describes burden; less theorised on functional engagement	Identifies regulatory process and structural preconditions, not only costs
Emotion regulation (Gross; Decety & Jackson)	Mechanisms for modifying emotional experience and expression	Not specific to perioperative clinical or team context	Applies regulatory science to perioperative demands
Burnout/compassion fatigue (Maslach; Figley)	Downstream adverse outcomes of sustained demand	Reactive outcome models; individual‐level intervention logic	Specifies antecedent regulatory process; locates determinants at system level
Calibrated emotional engagement (present model)	Dynamic, professionally regulated adjustment of emotional, attentional and relational involvement	Conceptually developed; requires empirical validation	Integrates process, context and SPO framing in perioperative nursing

*Note:* The final row summarises the present model.

Abbreviation: SPO, structure–process–outcome.

We introduce calibrated emotional engagement as the overarching theoretical concept, with calibrated empathy serving as a clinically accessible shorthand for the dimension most directly implicated in the patient relationship. The broader term is retained because the model encompasses attentional regulation, relational orientation and team‐directed emotional responsiveness beyond empathy in the narrow sense. In the operating room, emotional engagement is not exclusively patient‐directed: it includes reading the team's psychological state, maintaining communicative function under procedural pressure, and contributing to collective situational awareness. Engagement is the more precise superordinate term.

The concept builds on Gross (1998) process model of emotion regulation, which distinguishes antecedent‐focused strategies, those that modify emotional experience before it is fully activated, from response‐focused strategies that modulate expression once emotion has arisen. Both can be understood as operative in perioperative practice. A clinician who shifts attentional focus during a technically demanding phase may be engaging antecedent regulation; one who maintains composed demeanour during a crisis while experiencing internal arousal may be engaging response‐focused regulation. Calibrated emotional engagement involves the skilled, contextually sensitive deployment of both, not the suppression of feeling, but its active management in the service of clinical function.

The concept also draws on Decety and Jackson's (2004) account of empathy as requiring the maintenance of self‐other distinction, the capacity to register another's affective state without losing the boundary between that state and one's own. This is what Benner and Wrubel (1989) describe as involved, skilled caring: genuine engagement with the patient's situation that is neither absorbed into it nor defended against it. The erosion of this distinction under sustained affective load, termed affective flooding by Decety (2010), may be understood as a predictable consequence of insufficient regulatory support, not a failure of character.

A final clarification: calibrated emotional engagement is a functional concept, not a normative one. It does not prescribe how much clinicians should care, or in what manner. It offers a way of conceptualising what is required for sustainable, high‐quality perioperative practice and what conditions may support or undermine it. The distinction matters because the model aims to resist the individualisation of emotional difficulty, to insist that what looks like personal failure of regulation is often a predictable response to structural conditions that make calibration hard.

### 3.2 The Process of Calibration: Cue, Appraisal, Regulation

A theory of calibrated emotional engagement must specify not only what the calibrated state looks like, but how it is achieved and maintained in practice. Drawing on Gross (1998) process model and the appraisal tradition in emotion research, we propose that calibration involves a recurring micro‐cycle of situational perception, appraisal and regulatory response.

Situational cues signal the emotional and relational demands of a clinical moment. In the operating room these include a patient's visible distress at induction, a shift in team tone, an unexpected clinical development, a moment of procedural silence. Many such cues are attenuated in the cue‐deprived OR environment: the anaesthetised patient cannot signal fear or discomfort, and sterile draping obscures bodily expression. Calibration here therefore requires active attentional investment, a deliberate sustaining of the cognitive representation of the person beneath the drapes, rather than effortful response to incoming signals.

Appraisal involves the clinician's interpretation of what the situation requires: how much emotional involvement is appropriate, what relational register the encounter calls for, what the team needs communicatively. This appraisal is shaped by experience and professional identity. It is not always a conscious deliberative process; in experienced clinicians it may operate largely tacitly, as a skilled perceptual orientation consolidated through years of practice.

Regulatory adjustment follows appraisal. Antecedent‐focused strategies: cognitive reappraisal, attentional deployment, situation modification, operate before emotion is fully activated. Response‐focused strategies: expressive modulation, boundary maintenance, operate once arousal has arisen. The skill of calibration lies in the contextually sensitive deployment of both.

Miscalibration may be recognised through internal signals such as affective flooding or numbness; relational signals such as patient distress unregistered or team climate deteriorating; and performance signals such as communication failures. Recognition itself may require organisational support: debriefing cultures, peer trust and psychological safety create the conditions in which miscalibration can be named and addressed.

### 3.3 Clinical Illustration

Consider an anaesthesia nurse meeting an anxious patient immediately before induction. The patient asks whether they will wake up again, while the list is already delayed and the team is preparing for a technically demanding procedure. A response characterised by under‐engagement may reduce the encounter to task completion: confirming identity, attaching monitoring and moving rapidly towards induction while the patient's fear remains only minimally acknowledged. A response characterised by over‐engagement may involve absorbing the patient's fear as one's own, carrying it into the procedure and experiencing difficulty reorienting to the next case. Calibrated emotional engagement lies between these positions. The nurse registers the patient's fear as clinically and ethically meaningful, offers brief but genuine reassurance, maintains attentional readiness for the induction sequence, and then reorients to the wider team and technical demands of the case. Whether this calibration is achievable is not determined by individual empathy alone. Staffing pressure, team familiarity, time compression and opportunities for recovery all shape whether the nurse can remain present without becoming either detached or overwhelmed.

## 4 The Three‐Zone Model

We propose a three‐zone framework along the dimension of emotional engagement, from severe under‐engagement to severe over‐engagement, with a calibrated zone of functional practice between them. The zones are not rigid categories but regions of a continuum, and movement between them is shaped by both individual and organisational factors. The model does not assert that the calibrated zone is a permanently correct state but that sustained displacement from it, in either direction, may carry costs to patient care, team function and clinician sustainability.

### 4.1 Under‐Engagement

Under‐engagement may be characterised by emotional and relational involvement falling below the threshold required for responsive clinical practice. Its markers include routinised, formulaic patient interactions; reduced sensitivity to distress signals; a tendency to reduce the patient to the procedural task; and diminished connection to the meaning of clinical work. In the OR, under‐engagement may manifest as treating induction and emergence as procedural sequences rather than relational moments, as steps to be completed rather than encounters with a frightened person.

It is essential to resist a normative reading of under‐engagement as individual moral failure. It frequently represents a rational adaptive response to sustained high demand, a resource‐conserving strategy with short‐term functional value but cumulative clinical and relational costs. A clinician working in chronically understaffed conditions who reduces emotional exposure is not failing professionally; she is deploying a form of cognitive load management in the only way available to her. The source of the problem is the system. This aligns with Maslach (2001) account of depersonalisation as an adaptive response to chronic demand, and with the emotional labour literature's insight that the costs of caring work are generated by its conditions, not merely by its content (James 1992; Smith 1992). The clinical consequences of sustained under‐engagement are nevertheless significant: communication failures, missed patient cues and deterioration of team climate are associated with emotional disengagement in surgical environments (Flin et al. 2008).

### 4.2 Over‐Engagement

Over‐engagement may be characterised by emotional involvement that exceeds regulatory capacity for functional clinical performance. Its markers include difficulty separating from a patient's situation after a case ends; absorbing the patient's distress as one's own rather than registering it as clinical information; experiencing emotional flooding during high‐stakes moments; and persistent intrusive recollection. It is important to distinguish over‐engagement from strong caring orientation: the difficulty arises not from caring deeply but from affective absorption without adequate regulatory capacity, which may become a clinical liability under load.

The perioperative‐specific risk is partly structural. OR work involves repeated, compressed encounters with patients at their most vulnerable, often unconscious, invasively exposed and frightened at induction, generating high affective demand per unit time even when individual patient contact is brief. Accumulated without adequate recovery structure, this demand may produce conditions for over‐engagement even in clinicians with well‐developed regulatory capacity. When self‐other distinction becomes porous, the quality of clinical judgement may be compromised, a mechanism Decety (2010) terms affective flooding. This is a systemic outcome, not a personal one.

### 4.3 The Calibrated Zone

The calibrated zone is not a fixed emotional midpoint but a dynamic range of professionally functional engagement, continuously adjusted in response to patient need, procedural demands, and team context. Its markers include genuine present‐focused attention to the patient without affective absorption; the capacity to register emotional signals as clinical information rather than as stimuli demanding immediate response; and the ability to close a case and open the next without carrying the emotional residue of the former.

The calibrated zone is not characterised by emotional neutrality. A clinician operating within it may feel moved by a patient's situation, may experience concern, care, even grief. What distinguishes calibrated engagement is not the absence of feeling but the maintenance of self‐other distinction: the capacity to hold the patient's emotional reality alongside, rather than instead of, one's own regulated professional state. This is the third possibility that binary accounts of emotional protection, either detachment or over‐involvement, fail to capture. It is the kind of presence that Nortvedt (2001) describes as ethically responsive: genuinely affected, yet not overwhelmed.

The calibrated zone is also relationally extended beyond the patient dyad. In perioperative settings, it encompasses team‐directed responsiveness: reading colleagues' states, contributing to psychological safety, and communicating under pressure in ways that are functional rather than dysregulated. The quality of non‐technical skill performance in surgical teams, including communication, situational awareness and leadership, may be substantially dependent on this collective emotional climate (Flin et al. 2008; Gillespie et al. 2007). Calibrated engagement is not only a patient‐care competency; it is an organisational one.

**Figure 1:**
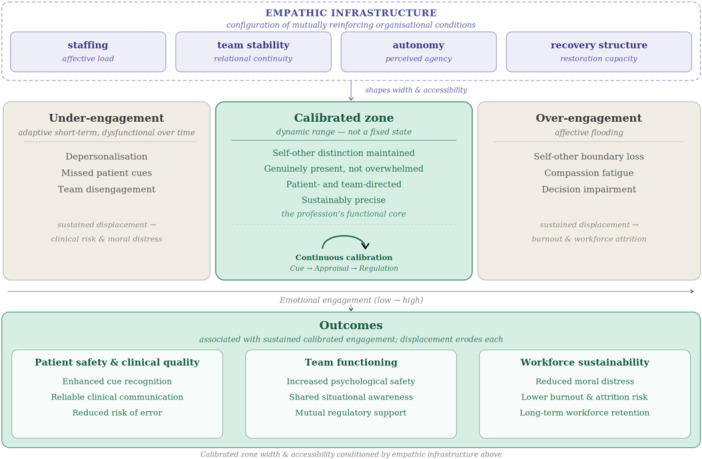
The three‐zone model of calibrated emotional engagement in perioperative care. The three zones represent positions along the emotional engagement continuum that result from the calibration process described in the text: the ongoing cycle of situational cue, appraisal and regulatory adjustment through which clinicians maintain, lose, or re‐enter the calibrated zone. Organisational conditions, including staffing, team stability, autonomy and recovery structure, shape the width and accessibility of the calibrated zone, and therefore condition whether the calibration cycle can be completed and sustained. Position within or displacement from the calibrated zone is associated with implications for patient safety, team functioning and workforce sustainability.

The calibrated zone varies in both width and accessibility across clinicians and contexts. Width refers to the breadth of the emotional range within which functional practice can be sustained: a wide calibrated zone can accommodate strong affective responses without displacement, whereas a narrow zone may be more easily disrupted. Accessibility refers to how readily a clinician can enter and re‐enter the zone following displacement. Both dimensions are shaped by individual capacity and by the organisational conditions described in the following section. This is why Figure 1 places organisational conditions above the zone model: they do not determine where a clinician falls on the continuum at any given moment, but they constrain and enable the width of the calibrated zone and the ease with which it can be accessed.

Although the calibrated zone is presented as a functional rather than moral ideal, the concept inevitably carries normative implications by identifying some forms of engagement as more clinically sustainable than others. Temporary deviation is not inherently problematic: short periods of emotional narrowing may be adaptive during acute crises, and strong affective involvement may be ethically meaningful after a difficult case. The concern is not momentary deviation but sustained displacement without recovery or structural support.

What counts as calibrated is also role‐ and context‐dependent. For an anaesthesia nurse at induction, calibration may require explicit relational presence and close attentional orientation to the patient's fear; for a scrub nurse during a technically demanding phase, it may require narrowed attentional focus with temporarily reduced emotional expression; for the wider team in the aftermath of an adverse event, it may require collective acknowledgement rather than rapid emotional closure. The model therefore does not define a single emotional style as ideal for all perioperative roles or situations. Rather, it asks whether deviations from the calibrated zone are situationally functional, ethically defensible and followed by sufficient recovery or reorientation to prevent sustained displacement. A further source of variation is career stage: a novice clinician may have a narrower and less readily accessible calibrated zone, particularly under acute demand, whereas experienced clinicians may have consolidated broader and more stable regulatory repertoires through accumulated practice. This variation is therefore an expected feature of calibrated emotional engagement rather than a deviation from the model.

## 5 Empathic Infrastructure: The Organisational Conditions of Calibration

The three‐zone model would reproduce the individualisation it aims to critique if it stopped at the level of individual regulatory capacity. The critical move is to ask: what determines whether calibrated engagement is achievable? We propose that this question has a substantially organisational answer, and that the four conditions described below may be understood as constituting an empathic infrastructure, the organisational substrate that makes calibrated emotional engagement possible in practice. The term does not denote a separate domain but serves as a heuristic to foreground the emotional conditions of clinical work within existing organisational structures.

The four domains described here are not intended as an exhaustive taxonomy of all organisational influences on emotional regulation. Rather, they are proposed as core domains because they map onto four immediate requirements for calibration: manageable affective load (staffing), relational continuity (team stability), perceived agency (professional autonomy) and restoration after emotional demand (recovery structure). Other structural factors, including authority gradients, interprofessional power dynamics and institutional cultures around emotional expression, undoubtedly also shape calibration and warrant further theoretical development.

These domains should not be read as independent levers. Their effects are likely cumulative and interactive. Staffing sufficiency shapes affective load directly, but also determines whether recovery opportunities are real rather than nominal: a rest break during an understaffed list is not the same resource as one during an adequately staffed list, because the conditions for psychological detachment differ fundamentally. Team stability supports relational continuity, but its regulatory value depends on whether authority gradients permit concerns, strain, or miscalibration to be voiced; a stable team in which hierarchy suppresses disclosure provides weaker buffering than one in which psychological safety is genuinely established. Professional autonomy may enable antecedent‐focused regulation, but only when workload and team climate allow discretionary judgment rather than forcing reactive suppression. Recovery structure can help clinicians re‐enter the calibrated zone after displacement, but it cannot fully compensate for chronically excessive load or unstable team configurations. Empathic infrastructure is therefore best understood as a configuration of mutually reinforcing conditions rather than a checklist of discrete resources: its regulatory value derives from how the domains operate in combination, and the absence of any single domain may significantly constrain what the others can achieve.

Staffing sufficiency is the most direct candidate. Under chronic understaffing, cognitive and affective load per clinician is high, within‐day recovery opportunities are eliminated, and the adaptive pressure towards under‐engagement is intensified. This may be understood as a patient safety issue as well as a workforce issue: the consequences for communication quality and error recognition are well documented in perioperative safety research.

Team stability, the degree to which perioperative teams work together consistently, shapes the interpersonal dimension of calibration. Stable teams may develop shared regulatory cultures: implicit understandings of how emotional work is distributed, tacit recognition of when a colleague is approaching the zone boundaries, and collective capacity to compensate under pressure. In high‐turnover or ad hoc configurations, this shared resource is absent. The evidence on team familiarity in surgery suggests that it affects not only efficiency but also outcomes: Witmer et al. (2022) found in a systematic review that operative team familiarity was associated with improved metrics across efficiency, patient safety and team satisfaction. The move towards flexible staffing models in perioperative nursing, motivated by cost‐efficiency, may systematically deplete this resource.

Professional autonomy, understood in the sense developed by self‐determination theory as the experience of volition and endorsement in one's work (Deci and Ryan 2000), may shape the quality of regulatory strategy available. Clinicians with high autonomy may be more likely to use active, antecedent‐focused regulation; those in low‐autonomy environments may tend towards suppression and reactive coping. Gunnarsdóttir et al. (2009) found in a large nursing study that front‐line management, staffing adequacy and nurse–doctor relationships were significant predictors of both nurse emotional exhaustion and patient outcomes, a pattern consistent with the claim that autonomy‐supportive organisational conditions function partly as empathic infrastructure. In perioperative teams, however, autonomy is not distributed evenly, but is shaped by authority gradients and interprofessional hierarchies that may affect whether concerns, emotional strain, or miscalibration can be voiced.

Recovery structure, comprising formal and informal opportunities for within‐session and between‐session psychological recovery, determines whether the calibrated zone can be re‐entered after displacement. This includes rest breaks and shift patterns but also team debriefing, peer support and institutional recognition that emotional work is work. Sagherian et al. (2023) found that nurses' rest breaks reduced fatigue only when they permitted genuine psychological detachment, and that workload level moderated this effect, suggesting that recovery structure is not simply a scheduling matter but a condition of whether recovery is functionally achievable. Where these structures are absent, the cumulative cost of demanding cases is borne privately. The absence of recovery structure is not a neutral organisational feature; it may be understood as an active redistribution of the costs of emotional labour onto individual clinicians, and thus as a policy choice with direct consequences for care quality.

Framed within Donabedian (1988) structure‐process‐outcome model, empathic infrastructure belongs to the structural domain: the conditions within which clinical processes occur. Calibrated emotional engagement is a process, a form of professional practice instantiated within those structural conditions. Patient safety, team functioning and workforce retention are downstream outcomes. The model implies that investment in empathic infrastructure may be usefully reframed as investment in clinical quality, not as a soft supplement to technical practice, but as one of its preconditions.

This argument also aligns with organisational research showing that staff support, management practices and wider care cultures are directly implicated in patient experience, safety and quality of care (Dixon‐Woods et al. 2014).

The Donabedian framework is used here not as the theoretical centrepiece of the model but as an organising heuristic that makes the relationships between empathic infrastructure, calibrated engagement and outcomes analytically explicit: structure shapes process, process generates outcomes and investment in structural conditions is therefore investment in clinical quality.

### 5.1 Implications for Practice, Education and Research

For workforce policy, the most immediate implication is a reorientation of where improvement effort is directed. If calibrated emotional engagement is substantially shaped by empathic infrastructure, then individual‐level interventions such as resilience training, mindfulness programmes and employee assistance address consequences rather than causes. This is not an argument against psychological support for nurses in distress; it is an argument that such support, in the absence of structural attention, cannot by itself sustain a workforce. Staffing ratios, team stability and recovery provision warrant consideration as patient safety investments, not only workforce wellbeing measures.

Operationally, this may involve staffing models that account for affective as well as technical workload; rostering practices that preserve team continuity during high‐intensity operating lists; and structured opportunities for brief debriefing after emotionally demanding cases. These measures are not presented as simple or resource‐neutral interventions, but as examples of how empathic infrastructure might be translated into organisational practice. Such translation will require engagement with institutional constraints, competing priorities and the practical realities of perioperative staffing, factors that the present model does not dissolve, but that it may help to name and address with greater theoretical precision.

For perioperative education, the model suggests that emotional regulation could be introduced as a core clinical competency, teachable, assessable and professionally significant, rather than treated as a pastoral afterthought. The content of such education would draw on emotion regulation science and reflective nursing practice: the cultivation of cognitive reappraisal, the development of self‐other differentiation under load, and an understanding of the organisational conditions that support or undermine calibration. Critically, it would include explicit attention to empathic infrastructure, so that clinicians understand their emotional responses not only as psychological events but as predictable consequences of work organisation, and can advocate accordingly.

For research, the concept opens empirical directions not well served by existing burnout metrics. These include the development of perioperative‐specific measures of emotional regulation quality sensitive to within‐day variation; studies of whether and how staffing, team stability and autonomy predict regulatory outcomes; and encounter‐level investigation of the relationship between clinician emotional calibration and patient experience at induction and emergence. The concept also invites integration with the non‐technical skills literature (Flin et al. 2008; Gillespie et al. 2007), which has not yet theorised the affective dimension of surgical team performance as a distinct and measurable construct.

## 6 Discussion: Reframing the Problem

This paper has proposed calibrated emotional engagement as a conceptual framework for understanding emotional regulation in perioperative care. Before concluding, it is worth being explicit about what the framework contributes and what it argues against. Existing frameworks describe emotional states such as burnout and compassion fatigue, capacities such as empathy, or individual strategies such as emotion regulation. The present model specifies the dynamic calibration process as it operates under real clinical conditions, identifies its organisational determinants, and proposes that sustainable emotional practice is as much a function of system design as of individual skill. The novelty claimed here is therefore not that emotional regulation, emotional labour, or organisational conditioning are new concerns in nursing, but that their interaction is specified, in the perioperative context, as a calibration process shaped by identifiable structural preconditions.

### 6.1 Individual Agency Within Structural Conditions

A potential misreading of the model is that it positions clinicians as passive respondents to structural conditions, with individual agency dissolved into organisational determinism. This is not the intent. Calibrated emotional engagement is individually enacted: it requires skill, experience and professional judgement that clinicians develop and exercise. What the model insists is that this enactment is always situated within structural conditions that expand or constrain what is possible. Individual skill and structural support are not alternatives; they are mutually constitutive.

The multilevel character of the model lies not simply in placing individual, team and organisation side by side, but in specifying how these levels condition one another. Individual regulatory skill shapes how situational cues are appraised and what strategies are available; team processes determine whether those appraisals can be voiced, shared, or collectively corrected; organisational design determines the load, continuity, autonomy and recovery through which individual and team regulation become either sustainable or progressively depleted. A highly skilled clinician may compensate for adverse conditions for a time, and a stable, cohesive team may buffer individual strain and miscalibration, but neither can indefinitely substitute for adequate structural support. At sufficient levels of organisational load, even strong teams lose their capacity to function as regulatory buffers, a dynamic this model conceptualises as one of its core failure modes. Conversely, structural improvement alone will not automatically produce calibrated engagement: clinicians and teams also require the skills, norms and psychological safety needed to use that infrastructure. The model's intervention logic is therefore combined rather than hierarchical: education, team development and organisational redesign are complementary levels of the same regulatory system, and investment at any single level without attention to the others is likely to yield diminishing returns.

Against the normative tradition, the model suggests that empathy is not a moral constant, a disposition that nurses either cultivate or fail to, but a regulated practice whose achievability depends on the conditions of work. The implication is not that empathy is unimportant: Benner and Wrubel (1989) are right that skilled caring is constitutive of good nursing, not incidental to it. The implication is that the instruction to ‘be more empathic’ is, without structural support, both individually unfair and clinically unreliable.

Against the burnout literature, the model proposes a shift in analytical focus: from tracing the pathway by which caring erodes over time, to specifying what functional regulation looks like in practice and what structural conditions make it achievable. Burnout frameworks capture important outcomes. Calibrated emotional engagement attempts to specify the regulatory processes that precede those outcomes, and to locate their key determinants in the organisation of work. This is not a trivial difference: it shifts the intervention logic from treating damaged individuals to designing supportive systems, a shift that the emotional labour tradition has long argued for (James 1992; Liaschenko and Peter 2004), but that has not yet been operationalised in perioperative nursing.

The model also intersects with scholarship on moral distress. Moral distress has commonly been linked to situations in which clinicians recognise an ethically appropriate course of action but are structurally or hierarchically constrained from acting accordingly, although the concept has itself been subject to considerable definitional debate (Morley et al. 2019). Calibrated emotional engagement does not replace moral distress as a concept, but may help specify one mechanism through which ethically constrained work becomes emotionally consequential. When clinicians repeatedly perceive patient or team needs that cannot be adequately addressed because of time pressure, hierarchical constraint, staffing insufficiency, or insufficient recovery structure, calibration may become progressively harder to sustain. In such circumstances, miscalibration is not simply emotional dysregulation; it may also reflect the accumulation of ethically loaded constraint within the organisation of care.

### 6.2 Scope and Transferability

The model is developed in relation to perioperative nursing, and its specificity to this context is a deliberate feature rather than a limitation. The characterisation of the OR as a cue‐deprived environment, the compressed encounter structure, the demands of anaesthetic induction and emergence and the team‐based nature of surgical work all shape the theory's content and give it its clinical purchase.

At the same time, the underlying argument, that emotional regulation is a multilevel process conditioned by organisational structure, may have applicability across other high‐demand clinical environments, including intensive care, emergency medicine and community nursing. The specific form of empathic infrastructure, the cues that trigger calibration, and the nature of team‐mediated regulation will differ across contexts. In intensive care or emergency nursing, calibration may be shaped by different relational cues: longer narrative continuity, family presence, repeated encounters over time, or acute unpredictability that takes a different form from the procedural compression of the OR. The perioperative version proposed here is specifically organised around anaesthesia, sterile draping, compressed relational exposure, rapid role transitions and dependence on surgical team coordination. These features do not make the model non‐transferable, but they do mean that application to other settings would require contextual re‐specification rather than straightforward adoption. Future work might usefully examine how the framework translates and where contextual specificity limits transferability.

The model has limitations that are those inherent in theoretical and conceptual work. It is offered as a provisional framework intended to generate testable propositions rather than to settle empirical questions: its value will ultimately be demonstrated through encounter‐level observation, interview‐based study and comparative organisational research rather than through conceptual argument alone. It is not intended as an empirical synthesis but as a conceptual framework that integrates existing strands of evidence into a new organising structure. The three zones are conceptually specified but not empirically validated. The organisational conditions identified as empathic infrastructure are theoretically grounded but their relative weight and interactions require empirical investigation. The concept is developed specifically in relation to perioperative nursing, though the underlying argument, that emotional regulation is organisationally conditioned, may have broader applicability across high‐demand clinical environments. Future work should attend to operationalisation, measurement and research designs capable of capturing the variation of calibrated engagement across different work environments and health system contexts. A more exhaustive theoretical engagement, with feminist accounts of care, poststructuralist accounts of professional identity, or the sociology of healthcare work, would enrich and complicate what is offered here as a conceptual foundation. The invitation to extend and contest the model is part of its purpose.

## 7 Conclusion

Empathy is not a moral constant. In high‐demand clinical environments it may be better understood as a regulated practice, one embedded in and enabled or constrained by the systems within which care is delivered. Calibrated emotional engagement offers a way of conceptualising this regulation in perioperative care: specifying its dimensions, its failure modes, and the organisational conditions that shape its achievability. The three‐zone model provides a clinically recognisable account of emotional states in the operating room, comprising under‐engagement, over‐engagement and the calibrated zone of functionally sustainable engagement, that neither idealises caring nor pathologizes its costs.

The person beneath the drapes is unconscious, exposed and trusting. Sustaining the capacity to remain genuinely present to that encounter, neither detached nor overwhelmed, neither cold nor consumed, is among the most demanding and least recognised aspects of perioperative professional practice. It is also, this paper has argued, a capacity that depends as much on how work is organised as on who the clinician is.

If perioperative systems are to be safe, sustainable and humane, they may need to be designed to support calibrated emotional engagement, not as a soft supplement to clinical practice, but as one of its structural preconditions. The concept of empathic infrastructure is an invitation to hold organisations accountable for the emotional conditions of care.

## Funding

The author has nothing to report.

## Ethics Statement

The author has nothing to report.

## Conflicts of Interest

The author declares no conflicts of interest.

## AI Disclosure

No generative AI/LLM was used in the production of this manuscript.

## Data Availability

Data sharing is not applicable to this article as no datasets were generated or analysed during the current study.
